# Pesticide Use, Perceived Health Risks and Management in Ethiopia and in Hungary: A Comparative Analysis

**DOI:** 10.3390/ijerph181910431

**Published:** 2021-10-03

**Authors:** Roba Argaw Tessema, Károly Nagy, Balázs Ádám

**Affiliations:** 1 Department of Public Health and Epidemiology, Faculty of Medicine, University of Debrecen, 4000 Debrecen, Hungary; roba.argaw@med.unideb.hu (R.A.T.); nagy.karoly@med.unideb.hu (K.N.); 2 Doctoral School of Health Sciences, University of Debrecen, 4000 Debrecen, Hungary; 3 Department of Environmental Health Sciences, College of Health and Medical Sciences, Haramaya University, Harar P.O. Box 235, Ethiopia; 4 Institute of Public Health, College of Medicine and Health Sciences, United Arab Emirates University, Al Ain 999041, United Arab Emirates

**Keywords:** pesticide, exposure, knowledge, risk perception, risk management, extension officer, plant doctor

## Abstract

Pesticides play a very important role for ensuring food security and economic growth but their use can cause harmful effects to human health and to the environment. The study aimed to investigate the level of knowledge, health risk perceptions, and experiences on the practice of pesticide use and management among extension officers in Ethiopia and plant doctors in Hungary. A questionnaire survey among 326 officers was conducted in the two study areas and data were analyzed by ordinal logistic regression. According to the findings, Hungarian officers had much better knowledge of pesticide products (92%), and less frequently experienced pesticide poisoning among applicators (7%) than the Ethiopians (66% and 41%, respectively). Hungarian officers perceived less health risk of pesticide use (AOR = 0.46, 95%, Cl: 0.27–0.80), were ten times more likely to deem the pesticide management system effective (AOR = 10.23, 95%, Cl: 5.68–18.46) and were nine times more likely to report that applicators used personal protective equipment (AOR = 8.95, 95%, Cl: 4.94–16.28). A significant proportion of officers from both countries reported inappropriate methods of pesticide residue disposal. These observations point out that the situation of pesticide use and knowledge and management of pesticide products is definitely better in Hungary; nevertheless, the issue continues to need more attention in both settings.

## 1. Introduction

Pesticides play an integral role in today’s farming. However, indiscriminate and incorrect use can be disastrous for human health and the environment all over the world. Pesticide poisoning is a global public and occupational health problem and accounts for nearly 300,000 deaths worldwide every year, the majority of which are from developing countries [1,2]. Human exposure to pesticides can occur in many ways: through occupational exposures, dietary residues, ambient outdoor and indoor exposures, contamination of drinking water, and unsafe use of pesticides on domestic animals [3]. Poison control centers data indicate that from all types of accidental poisoning, pesticides are responsible for 3.6% of adult and 3.4% of pediatric deaths with an overall proportion of 3.3% [4]. Acute pesticide poisonings have been reported as a major consequence in the farming community and the extent of poisoning in these workers, particularly in less developed countries, has often been based on inadequate information [5]. Adequate knowledge of pesticide health risks, proper attitude towards pesticide use, adequate storage, and handling of pesticides with appropriate application of a full set of personal protective equipment, are crucial to prevent morbidity and mortality from pesticide exposure. A study conducted in the central-eastern part of Ethiopia in 2016 indicated that the average annual pesticide use per hectare was 251 kg of active ingredients [6] and the prevalence of self-reported symptoms attributable to acute pesticide intoxications was 26% in 2014 [7]. A study conducted on agricultural workers in southeastern Hungary in 1998 showed that 22.6% of agricultural workers exposed to various complex mixtures of pesticides experienced pesticide poisoning and 12% of them had been hospitalized [8].

The main task of the extension workers in Ethiopia and plant doctors in Hungary (together called as officers) is to ensure safe use of pesticides. The extension workers’ duties range from advice to the individual farmers to adopt self-protective behavior to the farming companies to proper handling, effective and safe use of agrochemicals including pesticides in their control area. New knowledge and improved technologies are continuously transferred to the end users through extension officers [9,10]. The effectiveness of extension service delivery is critically depending on the knowledge of the officers on the various agricultural regulations and innovations they disseminate to the farmers. Practically, extension officers are the primary source of life-saving information; however, in many cases they may not have adequate training or skills and may have misperceptions about the risks of pesticides and their long-term impact on human health and the environment, so that they have a limited capacity to provide adequate services to farmers [11,12,13,14]. Most studies investigated farmers’ knowledge, perceptions, and attitude related to the health risks of pesticides but scanty of studies focused on extension officers (the other most critical actors in this context) who are supposed to be both theoretically and practically expert in providing appropriate advice to farmers and promote proper and safe handling practices of pesticides.

This study, provides a base for international comparison of information gained from officers responsible for ensuring adequate use of pesticides by applicators. A study on experiences about pesticide use, perceptions about the risks attributed to pesticides, knowledge regarding pesticides and pesticide management systems are important for several reasons. The officers’ perceptions directly influence the practice in the field. When the officers’ knowledge is insufficient, it may negatively influence farmers’ decisions regarding pesticide use and may lead farmers to take more risks than necessary. Hence, this study was designed to assess extension officers’ knowledge, attitude related to health risks of pesticides, experiences about pesticide use and management systems in Ethiopia and in Hungary and to forward applicable recommendations.

## 2. Materials and Methods

### 2.1. Study Design, Study Area and Source Population

A cross-sectional field study was conducted from 17 July to 24 August, 2019, in Ethiopia. In Hungary, data were collected through an interactive online survey platform from 16 September to 10 December 2020. Out of 27 districts in the east Hararge zone [15] and in the Harari region [16] in Ethiopia, six districts, namely Babile, Diretiyara, Haramaya, Karsa, Kombolcha, and Sofi were selected. In Hungary, thirteen counties, namely Baranya, Borsod-Abaúj-Zemplén, Fejér, Győr-Moson-Sopron, Hajdú-Bihar, Heves, Nógrád, Pest, Somogy, Szabolcs-Szatmár-Bereg, Vas, Veszprém, Zala, and the capital Budapest [17] were involved in the study. A total of 2462 plant doctors in the fourteen included areas geographically representative for Hungary and 754 agricultural extension workers in the five selected districts in Ethiopia served as the source population.

### 2.2. Sample Size and Sampling

The sample size for a statistical significance of results was determined by using a population proportion Formula (1) [18]):

n = [Nz^2^p(1 − p)]/[d^2^(N − 1) + z^2^p(1 − p)] (1)

where n = the sample size, N = the total number of extension officers in the study areas, z = the z value corresponding to 95% confidence level which is equal to 1.96, p = proportion of officers perceived a good pesticide related knowledge and competency, d = margin of error, desired precision, stated as a proportion of 0.05 at a 95% confidence level.

P was taken as 0.33 from a previous study in Ethiopia where 33% of the extension workers perceived good pesticide hazard related knowledge [14]; a 95% confidence interval (z = 1.96) and a 5% margin of error were considered. This gave a sample size of 234. In Hungary, p was taken as 0.93 from a similar study conducted in Georgia USA (2010), where 93% of extension officers perceived a good pesticide competency [19]; a 95% confidence interval and a 5% margin of error were considered. This gives a sample size of 96. 

In Ethiopia, the sampling consisted of three steps; six districts were selected based on the extent of pesticide use and agricultural activities out of 27 districts in the east Hararge Zone and Harari region. The probability proportional to size (PPS) sampling was used to calculate the sample size to each district after the total number of extension officers in six districts was identified. The systematic random sampling technique was used to select the study population. In Hungary, we had to continue the study using an interactive online survey platform due to the COVID-19 pandemic that broke out in the meantime. Every plant doctor in the involved areas was contacted by email asking for participation in the study.

### 2.3. Data Collection Procedure

In Ethiopia, data were collected by assisted questionnaire-based face-to-face interviews. Two well-trained individuals with previous experience in data collection were employed after training on how to administer the questionnaires. Field supervision was made by the principal investigator during data collection on a daily basis. In Hungary, due to the onset of the COVID-19 pandemic, field survey could not be conducted. Instead, a web-based survey using the Google Forms platform was applied. The survey link was administered by an invitation email asking for participation in the study. The web survey was deemed suitable for officers in Hungary because all of them had skills and access to the Internet and web technologies. Contact to officers was made through the county offices of the Hungarian Plant Protection Engineers and Plant Doctors’ Chamber, with the help of the Hajdú-Bihar County Office. An invitation letter containing the link to the online questionnaire was sent to each officer being a member of the Chamber. The purpose of the study and contact address of researchers in case of inquiry were explained at the beginning of the questionnaire for both study populations. 

### 2.4. Data Collection Instrument

The survey questionnaire was prepared in English and translated into Hungarian and Oromo, and then back to English to ensure its consistency. Data were collected using a structured and pre-tested questionnaire that comprised of 16 closed questions, some of them with open-ended part. The questionnaire collected information on three domains: demographic, knowledge and attitude, and experienced practice (Table 1). Questions related to socio-demographic characteristics as well as types of pesticides and disposal methods used multiple choice responses. To measure officers’ knowledge about pesticide products and routes of pesticide exposure, the study sought to evaluate whether officers were able to identify pesticides used by their name and mention the routes of exposure. The knowledge scores included: do you know pesticides by name? (scored 1 for yes; 0 for no response), mention as many pesticide products as you can (score 1 for less than 3; 2 for 3 and above responses), do you know the routes of pesticide exposure? (scored 1 for yes; 0 for no response), and list all the routes of entry (scored 1 for less than 3; 2 for 3 responses). These questions were scored by combinations of the answers an officer could give, and the total knowledge score was calculated. For both knowledge of pesticide products and of routes of exposure, the mean value (scored: 1.5) was used to dichotomize between the good (scored higher than 1.5) and poor (scored less than or equal to 1.5) knowledge category. For questions related to attitude and practices of pesticide use, management, and preventive measures, data were collected using multiple choice questions (MCQs) and a five-points psychometric Likers-scale, anchored from the lowest ordered extreme (1) to the highest ordered extreme (5): strongly disagree to strongly agree; very cheap to extremely expensive; ineffective to very effective; never or not at all to always, to measure the specific dimension of attitude and experiences of the officers.

### 2.5. Data Analysis

Data analysis was conducted using the SPSS Version 25 statistical package. Descriptive statistics, such as means, frequencies, and proportions, were calculated. Chi-square (X^2^) tests were used to assess the association between demographic factors, Knowledge and attitude to pesticide, practice of pesticide use and preventive measures with the nationality of the study subjects. In bivariate and multivariate analysis, ordinal logistic regression (OLR) model was used and adjusted to control for confounding factors and ascertain the independent predictors of the outcome variables. The OLR model was used because seven of the outcome variables had more than two ordered categories. Both crude odds ratio (COR) and adjusted odds ratio (AOR) were calculated to assess the strength of the association between outcome and explanatory variables. The significance of statistical associations was assured using odds ratios with 95% confidence interval (CI) and p-values. Statistical significance was accepted at the 5% level.

## 3. Results

### 3.1. Socio-Demographic Characteristics of Respondents

A total of 326 respondents (234 from Ethiopia and 92 from Hungary) have participated in the study. The majority (78%) of the respondents were male and most (43%) of them were 30 to 39 years old with a mean age of 38.4 (+9.5SD) years. A considerably higher proportion of the Hungarian participants belonged to the oldest 50-60 years age group (30% vs. 8%). On education, 87% of the Hungarian and 66% of the Ethiopian respondents had university degrees (BSc and above) (Table 2).

### 3.2. Types of Pesticides Used

Insecticides, herbicides, and fungicides were the most frequently reported types of pesticides used by agricultural workers in the study areas. The pesticides most frequently reported by Ethiopian officers were Malathion (85%), 2, 4-D (78%) and Diazinon (59%), while Glyphosate (97%), Deltamethrin (74%) and Pendimethalin (66%) were mainly reported by Hungarian officers. Based on the WHO classification, 70% of pesticides reported from Ethiopia and 60% reported from Hungary were moderately hazardous (WHO class II) (Table 3).

### 3.3. Knowledge of and Attitude to Pesticide Products

The study indicated that the Hungarian officers had better knowledge about pesticide products (92%) and routes of exposure (92%) than Ethiopian officers (66% and 58%, respectively), but less likely agreed that pesticides are toxic (51%), cause environmental pollution (61%) and use of pesticides carries risk for applicators (49%) than Ethiopian colleagues (74%, 82%, and 77%, respectively). In both countries, the majority of officers agreed that pesticides are extremely expensive (Table 4).

### 3.4. Practice of Pesticide Use, Preventive Measures and Pesticide Residues Disposal System

Fifty-seven percent of Hungarian and 14% of Ethiopian officers thought that the pesticide management system was effective; however, 83% of Hungarian and 46% of Ethiopian respondents reported illegal importation of pesticides from neighboring countries. Considerably more Ethiopian officers thought that farmers are rarely trained about the health risks of pesticides (81%), rarely used personal protective equipment (76%), and more often experienced pesticide poisoning among applicators in the past in their control area (41%) than the Hungarian responders (14%, 16%, and 7%, respectively) (Table 5).

**Figure 1:**
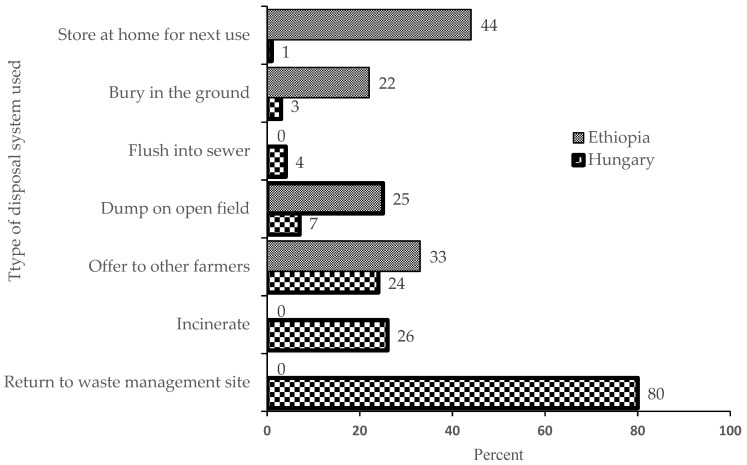
Pesticide residues disposal as reported by officers of the two study areas (Hungary *n* = 92; Ethiopia *n* = 234). Multiple choices were possible.

Regarding the pesticide residues disposal system, the majority (80%) of the Hungarian respondents agreed that agricultural workers returned the pesticide residues to a waste management site while most (44%) of the Ethiopian respondents reported that applicators stored pesticide residues at home for later use. In both study areas, a high proportion of the respondents testified that agricultural workers disposed pesticide residues improperly, through the situation was worse in Ethiopia (Figure 1).

### 3.5. Factors Determining Knowledge, Attitude and Practice Related to Pesticide Use

Nationality, sex, age, and education of respondents were used as the potential factors (explanatory variables) to identify their role in determining knowledge, attitude, and practice related to pesticide use (outcome variables). Nationality was found to be the strongest factor that significantly correlated with all aspects of knowledge, attitude and practice except the opinion on the cost of pesticides (Table 6 and Table 7). In addition, the knowledge about routes of pesticide exposure was found to be statistically significantly positively associated with the respondents’ educational status (β = 0.49, SE = 0.22, *p* = 0.026), indicating that more educated officers are more likely to have good knowledge about the routes of pesticide exposure. Also, officers’ attitude toward pesticide toxicity, opinion on the effectiveness of the pesticide management system were significantly positively and their opinion on the cost of pesticide products were significantly negatively associated with the respondents’ gender (β = 1.29, SE = 0.29, *p* < 0.001; β = 0.83, SE = 0.27, *p* = 0.002; and β = −0.58, SE = 0.27, *p* = 0.033, respectively), indicating that female officers are more likely to perceive that pesticides are toxic and the pesticide management system is effective but less likely to think that the cost of pesticide products is too high. Age had not shown a statistically significant association with any aspects of knowledge, attitude and practice related to pesticide use.

After controlling for sex, age, and education, nationality remained a significant independent predictor of all the ten aspects of knowledge, attitude, and practice related to pesticide use that showed a significant association in crude and adjusted analysis as well, indicating that the explanatory power of nationality is very strong. We found that Hungarian officers were less likely to think that pesticides are toxic (AOR = 0.55, 95%, Cl: 0.32–0.94, *p* < 0.05), cause environmental pollution (AOR = 0.47, 95%, Cl: 0.27–0.82, *p* < 0.01), and perceived less health risk of pesticide use among farmers (AOR = 0.46, 95%, Cl: 0.27–0.80, *p* < 0.01) (Table 8). Contrary, Hungarian respondents were over five times more likely to have good knowledge about pesticide products (AOR = 5.93, 95%, Cl: 2.42–14.55, *p* < 0.001) and routes of pesticide exposure (AOR = 5.78, 95%, Cl: 2.41–13.85, *p* < 0.001) compared to their counterparts. Conversely, the opinions of the officers in the two study areas were not significantly different about the cost of pesticide products.

Regarding practice, Hungarian officers were over 10 times more likely to deem the pesticide management system effective (AOR = 10.23, 95%, Cl: 5.68–18.46, *p* < 0.001), but over four times more likely to report illegal import of pesticide products from neighboring countries compared to their Ethiopian counterparts (AOR = 4.23, 95%, Cl: 2.16–8.31, *p* < 0.001) (Table 9). Hungarian officers were over 20 times more likely to think that farmers are well trained about the health risks of pesticides (AOR = 20.74, 95%, Cl: 10.61–40.57, *p* < 0.001), and 9 times more likely to report that farmers often use personal protective equipment (AOR = 8.95, 95%, Cl: 4.94–16.28, *p* < 0.001). Agricultural workers were 87% less likely to experience pesticide poisoning in the past in their control area than in Ethiopia (AOR = 0.13, 95%, Cl: 0.05–0.33, *p* < 0.001).

## 4. Discussion

This study investigates pesticides and their consequences in two countries, Hungary and Ethiopia, where agriculture has a similarly important historical role in the economy but which represents different levels of development. Based on the current study findings, a clear difference was observed in all aspect of knowledge, attitude, and experienced the practice of using pesticides between Ethiopian and Hungarian officers responsible for safe handling of these agrochemicals. A good level of knowledge of and risk attitude towards pesticide products among officers responsible for occupational safety and environmental health are vital for providing appropriate advice to reduce human health risks from inappropriate pesticide application among farmers [20]. The principal duty of extension workers is to transfer appropriate knowledge, skills, and attitude to the farmers so that farmers adopt self-protective behaviors, new and improved technologies, including pesticide use to increase productivity, while reducing risk from pesticide exposure [9,21,22].

In the present study, the Hungarian officers were older and had higher education than the Ethiopian extension workers, which is also reflected in their level of knowledge related to pesticides products and routes of exposure. This pronounced discrepancy may be attributed to the difference in socio-economic and demographic characteristics of study populations and more access to higher education in the case of Hungarian officers. The comparatively lower levels of knowledge about pesticide products and routes of pesticide exposure among Ethiopian extension workers impairs the safe use of pesticides by farmers in Ethiopia and negatively influences the adoption of self-protective behaviors. However, compared to the study done by Mormeta in the central part of Ethiopia, where only 33% of the extension workers perceived a good pesticide hazard related knowledge, it shows a promising improvement [14]. Nevertheless, more effort should be in place to address the knowledge deficit and to minimize the greater risk and vulnerability to the negative effects of pesticide exposure among Ethiopian applicators. Enhancing their level of education is crucial as the majority of them have college diplomas (attended two years education). The high level of knowledge and education among Hungarian officers, which is in line with findings of Cole in Georgia [19], and Remoundou et al. in Greece, Italy, and the UK [23], supports that education equips the officers with the ability to understand the very nature of pesticide products and convey accurate and uncensored information to the applicators in their working environment, and substantiates the need to provide continuous training to Ethiopian officers on various aspects of pesticide products and their management.

Insecticide products were more frequently reported to be used than herbicides and fungicides at a ratio of 7:3:3. According to the WHO hazard classification of pesticides, many reported pesticides pose a risk of moderate (e.g., 2,4-D, Diazinon, Deltamethrin, Dimethoate, Endosulfan, Sevin, DDT, Epoxiconazole) and slightly acute toxicity (e.g., Malathion, Glyphosate, Chloronitrile, Pendimethalin) [24]. Seventy-percent of pesticide products reported from Ethiopia and 60% reported from Hungary were moderately hazardous (WHO class II). Pendimethalin, Epoxiconazole, and Tetraconazole were reported only by Hungarian, while Malathion, Sevin, and DDT only by Ethiopian officers. Besides acute risk, some reported pesticides may have a tendency to bioaccumulate in the food chain (e.g., Deltamethrin, DDT, Endosulfan) posing chronic health risks from long-term low-dose exposure [25,26,27]. The toxic and persistent organic pollutant organochlorine pesticide products, like DDT, were reported from Ethiopia, Endosulfan, and even from Hungary, which had already been banned in most countries worldwide [28,29]. In Ethiopia, DDT is still actively sprayed for malaria vector control by the Ministry of Health but farmers also apply it to food crops and khat (Catha edulis) in illegal ways [30,31]. In addition to DDT, the persistent organochlorine Endosulfan had also been banned from the member states of the European Union, including Hungary [25].

Misperception regarding pesticide products can seriously impair the capacity of farmers to adopt self-protective behaviors against the risks of acute pesticide poisoning and long-term health effects. In this study, a substantial proportion of officers lessened the danger of pesticides; almost half (51%) of the Hungarian and nearly three-fourths (74%) of the Ethiopian officers agreed that pesticides are toxic. These findings are consistent with the observation of a study in Mexico where 75% of extension workers reflected with correct risk perception the inherent poisonous ability of a pesticide [32], and another where only a quarter of the extension workers perceived pesticides as a major problem in the community they served in Tanzania [11]. This triggers the necessity of intensive in-depth education and regular in-service training and capacity building, because this misperception of extension workers about the inherent poisonous ability of a pesticide influences the extent of decisions making to control risks and design appropriate interventions, and the amount and the quality of safety training and information they offer to the farmers [33,34,35,36].

In Hungary, the pesticide distribution, transportation, and storage system were deemed quite effective (74%), the policy coverage and the law enforcement on occupational and environmental health is much stricter, and the efforts made to govern the process seem well organized; however, a substantial proportion (83%) of officers reported that there is a practice of unregistered import of pesticide products from neighboring countries. Hence, this gives a signal that the current pesticide management system needs to be evaluated and strengthened. On the other hand, in Ethiopia, the pesticide management system appeared to be ineffective (56%), though the policy coverage is sufficient, the law enforcement on occupational and environmental health is poor, and the efforts made to govern the process are not well organized. The Ministry of Agriculture was found to be the largest pesticide distributor to farmers in Ethiopia; however, a considerable proportion of the respondents (46%) reported the practice of import of unregistered and illegal pesticide products from neighboring countries. The findings from both study areas indicate that there is a possibility for users to buy uncontrolled pesticide products directly from illegal sources, probably at a lower cost. A greater proportion of Hungarian officers thought that the way how currently pesticides are applied by users poses less risk to their health, and pesticide residues are less likely to be present in food, air, water, and soil in the environment than their Ethiopian counterparts. This finding is consistent with a study conducted in North Carolina where a substantial number of extension workers felt that users were at low risk of exposure because they received training and used protective equipment as required [33]. A study conducted in Saudi Arabia supports findings from Ethiopia where extension workers believed that pesticide residues are a significant health threat to our food and environment [37]. This discrepancy is probably due to the different level of effectiveness of preventive measures in place, which is determined by economic and social development status. A high proportion of Hungarian officers thought that farmers are fully trained about the health risk of pesticides and used the full set of necessary personal protective equipment during pesticide application. This may be the reason that Hungarian officers claimed lower health risk of pesticide use among farmers as they thought that the preventive measures were better and the pesticide management system is effective in their control area, since the health risk of pesticide use is determined by the combination of toxicity of pesticide and its exposure conditions.

A significant proportion of Ethiopian officers reported that they experienced pesticide-related poisoning and illnesses among farmers at some point in the past in their control area. This finding is clearly supported by the unsafe use of pesticides, poor utilization of PPE, inadequate training of farmers and officers, and signifies that the risk-awareness towards pesticides is high but preventive practices are still insufficient. Hence, the knowledge and skill transfer should be evaluated based on the real-life working conditions of the farmers in Ethiopia; otherwise, the quality of advisory service remains low, posing a threat to pesticide applicators and to the environment at large. Pesticide risk communication and safety education methods, which focus only on providing information, are inadequate, rather sustainable participatory pesticide safety approaches and behavioral change risk reduction strategies should be applied [36]. Advisory services provided by extension officers about pesticide application should be consistently evaluated during field spraying because in most cases farmers have a low level of education but usually well-recognize and adopt protective behaviors through learning-by-doing [32].

Eighty percent of officers in Hungary reported that farmers returned leftover pesticide products to waste management sites as the dominant method of disposal. This finding is promising and acknowledges the effort made by the sector, while a significant proportion of Ethiopian officers (44%) reported that farmers stored leftover pesticide residues at home for future use and/or offered to other farmers (33%), probably because their limited financial capacity. This practice may not only subject the farmers to high risk of pesticide exposure, but by take-home pathways also increase the risk of non-occupational exposure among family members, neighbors, and bystanders, as well as of the contamination of environmental compartments (air, water, soil, and food). In general, extension workers may tend to give more emphasis on higher product yields for farmers by increased use of pesticide products, and its side effects on human health and on the environment may have less weight [32,38]; though its long- and short-term effects are not only confined to the operators, but these exposures can reach their family members, including children and pregnant women, re-entry workers, bystanders, residents, and consumers at large through direct (dermal, inhalation, and ingestion) and indirect (through contaminated water, food, soil, and air) routes of exposure [23]. Hence, conducting studies on the health risk of pesticide exposure to the general public is very important to safeguard the whole community.

## 5. Limitations

This study has its potential limitations. First, the cross-sectional survey design pro-vides the characteristics of a population at one point in time about the use, knowledge, perceived health risks, and management practices of pesticides, which cannot make causal inference, and has a limited use to examine behavior. Further observational and experimental studies are required to detect the actual experiences and practices related to pesticide products. Second, the Hungarian study population was limited by the low participation rate; this is one of the drawbacks associated with online surveys using self-administered questionnaire, and may affect representativeness. However, the included areas are geographically well representative for Hungary. Finally, the information gathered by the questionnaire was self-reported, and therefore it is not supported by qualitative measurement within the study framework; hence, the response may over or under-estimate the factual practice, experiences, and perceptions of officers about pesticide exposure due to recall bias or self-reported bias.

## 6. Conclusions

This study observed clear differences in officers’ knowledge, attitude, and experienced practice of pesticide use in Ethiopia and Hungary. From the response of the officers, it can be concluded that there is a large difference in the effectiveness of preventive measures between the two study areas. Interventions improving extension services, overall optimization of the pesticide management system through strict law enforcement of occupational and environmental health regulations, and provision of adequate pesticide safety training, including process and content dimensions of pesticide use to ensure sufficient knowledge and skills for identifying and controlling the unnecessary pesticides exposure of farmers and the environment without compromising product yield, are recommended, especially in Ethiopia, where based on the information provided by this study, further investigation of pesticide exposure by directly assessing the real occupational health risks of pesticide applicators can be carried out.

## Figures and Tables

**Table 1:** Overview of the questions included in the questionnaire used in the study.

Domains	Descriptions
Demographic questions	Gender; age; education level, occupation, and nationality
Knowledge and attitude related questions	Knowledge about pesticide products; knowledge of routes of pesticide exposure; perception about pesticide toxicity; opinion about cost of pesticides; perception about environmental pesticide pollution; and perception about health risks of pesticides
Experienced practices related questions	Types of pesticides used; illegal import of pesticide products; pesticide management system, pesticide disposal system; training about health risks of pesticides, use of personal protective equipment; and experience of pesticide poisoning

**Table 2:** Socio-demographic characteristics of respondents in Hungary and Ethiopia.

Characteristics	Categories	N (%)	Hungary (*n* = 92)		Ethiopia (*n* = 234)		*p*-Value
			N	(%)	N	(%)	
Sex	Male	255 (78)	65	71	190	81	0.038
	Female	71 (22)	27	29	44	19	
Age	18–29	50 (16)	11	12	39	17	<0.001
	30–39	141 (43)	28	30	113	48	
	40–49	89 (27)	25	28	64	27	
	50–60	46 (14)	28	30	18	8	
Education	College diploma	91 (28)	12	13	79	34	<0.001
	University BSc	170 (52)	29	32	141	60	
	University MSc and above	65 (20)	51	55	14	6	

**Table 3:** Pesticides used as reported by officers in Hungary and Ethiopia.

Pesticide Type	Active Ingredient	WHO Toxicity Class	Chemical Group	Hungary N * (%)	Ethiopia N * (%)
Insecticide	Malathion	III	Organophosphate	-	199 (85.0)
Herbicide	2, 4-D	II	Phenoxy alkonates	19 (20.7)	182 (77.8)
Insecticide	Diazinon	II	Organophosphate	7 (7.6)	138 (59.0)
Herbicide	Glyphosate	III	Glycine derivative	89 (96.7)	114 (48.7)
Insecticide	Deltamethrin	II	Pyrethroids	68 (73.9)	69 (29.5)
Insecticide	Dimethoate	II	Organophosphate	24 (26.1)	67 (28.6)
Insecticide	Endosulfan	II	Organochlorine	4 (4.3)	61 (26.1)
Insecticide	Sevin	II	Carbamate	-	46 (19.7)
Fungicide	Chloronitrile	III	Chloronitrile	28 (30.4)	33 (14.1)
Insecticide	DDT	II	Organochlorine	-	13 (5.6)
Herbicide	Pendimethalin	III	Dinitroaniline	61 (66.3)	-
Fungicide	Epoxiconazole	II	Triazole	53 (57.6)	-
Fungicide	Tetraconazole	IV	Triazole	44 (47.8)	-

NB: WHO classification of pesticide by hazards (LD50): Ia = Extremely hazardous; Ib = Highly hazardous; II = Moderately hazardous; III = Slightly hazardous; IV = Unlikely to present acute hazard in normal use. * Multiple choices were possible.

**Table 4:** Knowledge of and attitude to pesticides among officers in Hungary and Ethiopia.

Characteristics	Categories	N (%)	Hungary (*n* = 92)		Ethiopia (*n* = 234)		*p*-Value
			N	(%)	N	(%)	
Knowledge about pesticide products	Good	239 (73)	85	92	154	66	<0.001
	Poor	87 (27)	7	8	80	32	
Knowledge about routes of pesticide exposure	Good	221 (68)	85	92	136	58	<0.001
	Poor	105 (32)	7	8	98	42	
Attitude toward pesticide toxicity	Disagree	31 (10)	11	12	20	9	<0.001
	Undecided	76 (23)	34	37	42	18	
	Agree	140 (43)	25	27	115	49	
	Strongly agree	79 (24)	22	24	57	24	
Opinion about the cost of pesticide products	Moderately expensive	33 (10)	13	14	20	9	0.309
	Very expensive	122 (37)	34	37	88	38	
	Extremely expensive	171 (53)	45	49	126	54	
Attitude toward environmental pesticide pollution (water, food, air, and soil)	Disagree	54 (17)	24	26	30	13	0.001
	Undecided	25 (8)	12	13	13	6	
	Agree	109 (33)	25	27	84	36	
	Strongly agree	138 (42)	31	34	107	46	
Perceived health risk of pesticide use	Disagree	64 (20)	29	32	35	15	<0.001
	Undecided	37 (11)	18	20	19	8	
	Agree	148 (45)	25	27	123	53	
	Strongly agree	77 (24)	20	22	57	24	

**Table 5:** Experienced practice of pesticide use and preventive measures among officers in Hungary and Ethiopia.

Characteristics	Categories	N (%)	Hungary (*n* = 92)		Ethiopia (*n* = 234)		*p*-Value
			N	(%)	N	(%)	
Effectiveness of the pesticide management system	Ineffective	142 (44)	11	12	131	56	<0.001
	Somewhat effective	68 (21)	13	14	55	24	
	Moderately effective	31 (9)	16	17	15	6	
	Effective	85 (26)	52	57	33	14	
Illegal import of pesticides	Yes	184 (56)	76	83	108	46	<0.001
	No	142 (44)	16	17	126	54	
Training of farmers about the health risk of pesticides	Rarely	202 (62)	13	14	189	81	<0.001
	Sometime	64 (20)	36	39	28	12	
	Fully trained	60 (18)	43	47	17	7	
Use of personal protective equipment (PPE) by farmers	Rarely	193 (59)	15	16	178	76	<0.001
	Sometimes	60 (19)	39	43	21	9	
	Very often	73 (22)	38	41	35	15	
Experience of pesticide poisoning among farmers	No	223 (68)	86	93	137	59	<0.001
	Yes	103 (32)	6	7	97	41	

**Table 6:** Factors determining knowledge of and attitude to pesticide use among officers in Hungary and Ethiopia.

Explanatory Variables		Outcome Variables					
		Knowledge about Pesticides Products	Knowledge about Routes of Pesticide Exposure	Attitude towards Pesticide Toxicity	Opinion about the Cost of Pesticide Products	Attitude towards Environmental Pesticide Pollution	Perceived Health Risk of Pesticide Use
Country (1 = Hungary, 2 = Ethiopia)	β	−1.78	−1.75	0.60	0.15	0.75	0.77
	SE	0.46	0.45	0.27	0.29	0.28	0.28
	*p*-value	0.000 ***	0.000 ***	0.028 *	0.614	0.007 **	0.006 **
Sex (1 = Male, 2 = Female)	β	0.214	0.10	1.29	−0.58	0.07	0.08
	SE	0.35	0.34	0.29	0.27	0.27	0.27
	*p*-value	0.543	0.773	0.000 ***	0.033 *	0.800	0.761
Age (1 = 18–29, 2 = 30–39, 3 = 40–49, 4 = 50–60)	β	−0.031	0.21	0.01	0.15	0.03	0.12
	SE	0.16	0.16	0.14	0.13	0.12	0.12
	*p*-value	0.850	0.195	0.912	0.249	0.803	0.353
Education (1 = diploma, 2 = BSc, 3 = MSc and over)	β	0.085	0.49	−0.25	−0.22	−0.01	−0.19
	SE	0.22	0.22	0.17	0.18	0.17	0.17
	*p*-value	0.703	0.026 *	0.143	0.216	0.941	0.257

NB: β = Regression coefficient, Beta; SE = Standard Error, the first category was used as reference for all explanatory variables. * *p* < 0.05, ** *p* < 0.01, *** *p* < 0.001.

**Table 7:** Factors determining practice of using pesticides among officers in Hungary and Ethiopia.

Explanatory Variables		Outcome Variables				
		Effectiveness of Pesticide Management System	Illegal Import of Pesticides	Training of Farmers about the Health Risk of Pesticides	Use of PPE by Farmers	Experience of Pesticide Poisoning among Farmers
Country (1 = Hungary, 2 = Ethiopia)	β	−2.33	−1.44	−3.03	−2.19	2.04
	SE	0.30	0.34	0.34	0.30	0.47
	*p*-value	0.000 ***	0.000 ***	0.000 ***	0.000 ***	0.000 ***
Sex (1 = Male, 2 = Female)	β	0.83	0.14	0.58	0.19	−0.55
	SE	0.27	0.31	0.30	0.29	0.36
	*p*-value	0.002**	0.656	0.055	0.500	0.120
Age (1 = 18–29, 2 = 30–39, 3 = 40–49, 4 = 50–60)	β	−0.10	0.02	−0.27	0.06	0.01
	SE	0.13	0.15	0.15	0.14	0.16
	*p*-value	0.444	0.885	0.071	0.642	0.936
Education (1 = diploma, 2 = BSc, 3 = MSc and over)	β	−0.13	0.39	−0.01	−0.17	−0.42
	SE	0.18	0.20	0.20	0.19	0.22
	*p*-value	0.474	0.053	0.967	0.371	0.057

NB: β = Regression coefficient, Beta; SE = Standard Error, the first category was used as reference for all explanatory variables. ** *p* < 0.01, *** *p* < 0.001.

**Table 8:** Effect of nationality on the knowledge of and attitude to pesticides in Hungary and Ethiopia.

Country	Outcome Variables (*n* = 326)				Odds Ratio [95%, CI]	
					Crude Odds Ratio [COR]	Adjusted Odds Ratio [AOR] †
	Knowledge about pesticide products					
	Poor		Good			
Hungary	7 (8)		85 (92)		6.31 [2.79–14.28] ***	5.93 [2.42–14.55] ***
Ethiopia	80 (34)		154 (66)		1.00	1.00
	Knowledge about routes of pesticide exposure					
	Poor		Good			
Hungary	7 (8)		85 (92)		8.75 [3.88–19.73] ***	5.78 [2.41–13.85] ***
Ethiopia	98 (42)		136 (58)		1.00	1.00
	Attitude toward pesticide toxicity					
	Disagree	Undecided	Agree	Strongly agree		
Hungarian	11 (12)	34 (37)	25 (27)	22 (24)	0.56 [0.36–0.88] *	0.55 [0.32–0.94] *
Ethiopian	20 (8)	42 (18)	130 (49)	42 (25)	1.00	1.00
	Opinion about the cost of pesticides products					
	Moderately expensive	Very expensive	Extremely expensive			
Hungary	13 (14)	34 (37)	45 (49)		0.77 [0.48–1.22]	0.86 [0.49–1.53]
Ethiopia	20 (8)	76 (38)	138 (54)		1.00	1.00
	Attitude towards environmental pesticide pollution					
	Disagree	Undecided	Agree	Strongly agree		
Hungary	24 (26)	12 (13)	25 (27)	31 (34)	0.48 [0.31–0.76] ***	0.47 [0.27–0.82] **
Ethiopia	30 (13)	13 (5)	84 (36)	107 (46)	1.00	1.00
	Perceived health risk of pesticide use					
	Disagree	Undecided	Agree	Strongly agree		
Hungary	29 (32)	18 (20)	25 (27)	20 (22)	0.44 [0.28–0.69] ***	0.46 [0.27–0.80] **
Ethiopia	35 (15)	19 (8)	123 (53)	57 (24)	1.00	1.00

NB:, * *p* < 0.05, ** *p* < 0.01, *** *p* < 0.001; † adjusted for sex, age and education; numbers enclosed in parentheses indicate the percentage of the respondents to each category of the outcome variables.

**Table 9:** Differences in practice of using pesticides in Hungary and Ethiopia.

Country	Outcome Variables (*n* = 326)				Odds Ratio [95%, CI]	
					Crude Odds Ratio [COR]	Adjusted Odds Ratio [AOR] †
	Effectiveness of the pesticide management system					
	Ineffective	Somewhat effective	Moderately effective	Effective		
Hungary	11 (12)	13 (14)	16 (17)	52 (57)	9.15 [5.57–15.04] ***	10.23 [5.68–18.46] ***
Ethiopia	131 (56)	55 (24)	15 (6)	33 (14)	1.00	1.00
	Illegal import of pesticides					
	No		Yes			
Hungary	16 (17)		76 (83)		5.54 [3.05–10.07] ***	4.23 [2.16–8.31] ***
Ethiopia	126 (54)		108 (46)		1.00	1.00
	Training of farmers about the health risk of pesticide					
	Rarely	Sometimes	Fully trained			
Hungary	13 (14)	36 (39)	43 (47)		17.86 [10.32–30.91] ***	20.74 [10.61–40.57] ***
Ethiopia	189 (81)	28 (12)	17 (7)		1.00	1.00
	Use of personal protective equipment by farmers					
	Rarely	Sometimes	Very often			
Hungary	15 (16)	39 (43)	38 (41)		8.27 [5.08–13.48] ***	8.95 [4.94–16.28] ***
Ethiopia	178 (76)	21 (9)	35 (15)		1.00	1.00
	Experience of pesticide poisoning among farmers					
	No		Yes			
Hungary	86 (93)		6 (7)		0.10 [0.04–0.24] ***	0.13 [0.05–0.33] ***
Ethiopia	137 (59)		97 (41)		1.00	1.00

NB: *** *p* < 0.001; † adjusted for sex, age and education; numbers enclosed in parentheses indicate the percentage of the respondents to each category of the outcome variables.

## Data Availability

Data from this study are available from the corresponding author on reasonable request.
